# Ecosystem Services Insights into Water Resources Management in China: A Case of Xi’an City

**DOI:** 10.3390/ijerph13121169

**Published:** 2016-11-24

**Authors:** Jingya Liu, Jing Li, Ziyi Gao, Min Yang, Keyu Qin, Xiaonan Yang

**Affiliations:** 1 College of Tourism and Environment, Shaanxi Normal University, Xi’an 710062, Shaanxi, China; liujingya0709@163.com (J.L.); m18535840577_1@163.com (Z.G.); yangxiaonan@snnu.edu.cn (X.Y.); 2 Key Laboratory of Disaster Monitoring and Mechanism Simulation, Baoji University of Arts and Sciences, Baoji 721000, Shaanxi, China; 3 College of Territorial Resources and Tourism, Anhui Normal University, Wuhu 241000, Anhui, China; ahnuyangmin@163.com; 4 Key Laboratory of Marine Geology and Environment, Institute of Oceanology, Chinese Academy of Sciences, Qingdao 266000, Shangdong, China; qdqky924@126.com

**Keywords:** Xi’an, ecosystem service, water resources management, survey, ecosystem service indicators

## Abstract

Global climate and environmental changes are endangering global water resources; and several approaches have been tested to manage and reduce the pressure on these decreasing resources. This study uses the case study of Xi’an City in China to test reasonable and effective methods to address water resource shortages. The study generated a framework combining ecosystem services and water resource management. Seven ecosystem indicators were classified as supply services, regulating services, or cultural services. Index values for each indicator were calculated, and based on questionnaire results, each index’s weight was calculated. Using the Likert method, we calculated ecosystem service supplies in every region of the city. We found that the ecosystem’s service capability is closely related to water resources, providing a method for managing water resources. Using Xi’an City as an example, we apply the ecosystem services concept to water resources management, providing a method for decision makers.

## 1. Introduction

Water resource shortages are becoming an increasingly common and serious global problem [1]. China’s population is predicted to increase to 1.6 billion people by 2030. The country’s water resources per capita is currently estimated at 2220 m^3^; however, these resources are projected to fall to 1760 m^3^ by 2030 [2]. Meeting water resource demands is a particular challenge in arid and semi-arid areas [3]. 

The rapidly accelerating pace of construction in the city of Xi’an and the Guanzhong-Tianshui Zone, has created one of the most significant water shortages in China. The water department in Xi’an is responsible for water management integration, and was established early in the city’s development [4]. The Department has deployed a variety of water management models in recent years, but none has fully adapted to the social and economic development [4]. Effective water resource management is of significant importance for the city’s sustainable development [5]. As such, in recent years, integrated water resources management (IWRM) has become a focal concern, especially in arid areas [6].

Ehrlich and Ehrlich proposed the concept of “ecosystem services” as a way to assess the impact and effectiveness of ecosystems on human society. Ecosystem services are “the conditions and processes through which natural ecosystems, and the species that make them up, sustain and fulfill human life” [7]. Ecosystem services were later divided into a four category classification scheme that is now widely accepted and used in the Millennium Ecosystem Assessment (MA) [8]. The four ecosystem service categories include: supply services, regulating services, cultural services, and support services. All of these categories are closely related to human activities [8]. 

Recent studies have investigated the tradeoffs and synergies between multiple ecosystem services. One study evaluated the trade-off between carbon sequestration and water yield [9]; another study compared the trade-off and synergy between water yield, agricultural production, soil conservation, water interception, and carbon sequestration [10]. Understanding how human interventions influence ecosystem service functions, and understanding how the functions impact human welfare is a significant challenge [11]. For example, one study investigated the effects of biomass harvesting on the capacity of the forest ecosystem to provide water and climate regulation benefits to local and global beneficiaries [12]. The close relationship among each ecosystem services provides opportunities to apply ecosystem services to natural resource management. The ecosystem service function has become an important factor that people need to consider in managing natural resources or formulating relevant policies [13], with the ultimate goal of maintaining ecological balance.

The relationship between ecosystem services and water resources management has been gaining increased attention, with some considering the concepts to be almost the same [14]. Some people have tried to use ecosystem services to also provide integrated water resources management services [15]; others have used ecosystem and water resources management when facing multiple stressors [16]. These activities have demonstrated that ecosystem services can support water resources planning, with mutually supportive opportunities. However, there is currently no mature water management framework in Xi’an, so the area continues to face water problems [17].

In this case study, we applied an ecosystem services framework to research water resource management. More specifically, we used an online survey (questionnaire) and an index analysis to study Xi’an ecosystem services. The questionnaire was used to gauge people’s understanding of ecosystem services, providing a score to measure each index’s weight [18]. This index analysis was used to study ecosystem service in the districts and counties that support water resources management [19]. We compared the correlation between ecosystem services and water resources in Xi’an, facilitating the construction of our framework. It does not currently have a mature ecosystem services framework to support integrated water resources management in China. However, many cases have demonstrated that reforming a systematic approach between ecosystem services and water resources may support water resources management [20]. For example, ecological footprints have been used to study water resources [21]. This research developed a new method for water management, effectively combining water resource management with ecological system management, so they both promote each other. Ecosystem services can support water resources management, and rational water resource management can further enhance ecosystem service. 

## 2. Methods

**Figure 1:**
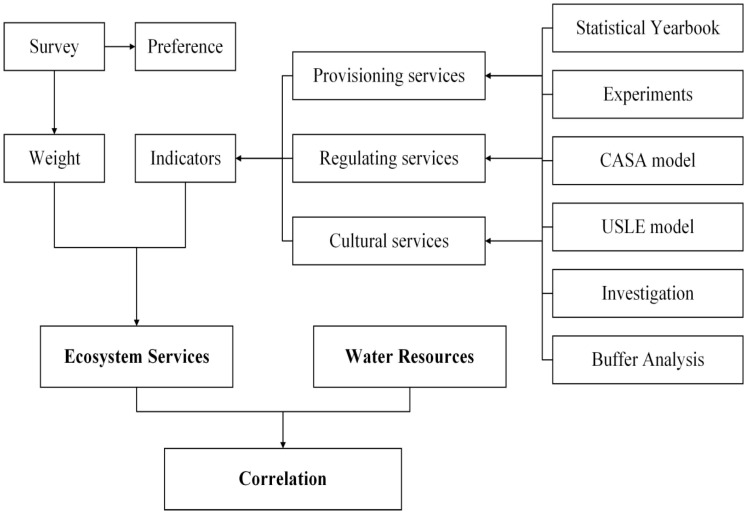
Flowchart.

In this paper, three kinds of ecosystem services are selected, and the cognition of people about ecosystem services is investigated through a questionnaire survey. The weight of each indicator is evaluated through a questionnaire survey of experts, and the supply ability of various ecosystem services functions is calculate through the Likert method. We have compared the supply capacity of ecosystem services and water resources in Xi’an, and found that there is a correlation between them, which provides a possibility for us to establish a new framework for water resource management (Figure 1).

### 2.1. Study Area

**Figure 2:**
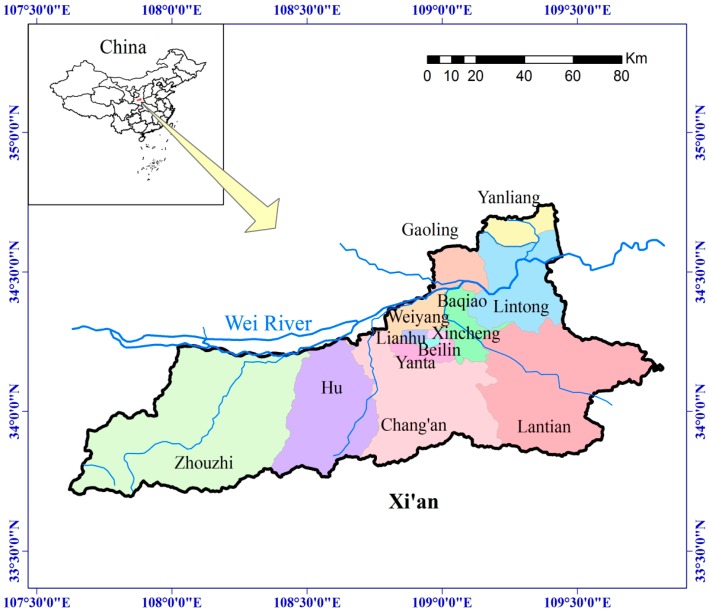
Study area location.

Xi’an is located in the center of China, and is the capital of Shaanxi Province (Figure 2). Xi’an has a warm-humid continental monsoon climate, and experiences all four seasons [22]. Xi’an has eight main rivers: Wei River, Jing River, Feng River, Lao River, Jue River, Hao River, Chan River, and Ba River. These rivers constitute the Xi’an drainage network, and they all belong to the Yellow River water system. The rivers share the common characteristic of having an uneven run-off distribution across time and space. The run-off during a high-flow year is 4–7 times greater than during a low-flow year, and the annual run-off in some mountain areas can vary by 10 times. In a low-flow year, some rivers run dry. The annual fluctuation of run-off is also significant. For example, only 2% of the annual run-off generally occurs during the dry season month of February, while 45%–56% of the total annual run-off generally occurs during the flood season. Natural and socio-economic factors result in Xi’an being a city with severe water shortages, posing an imminent problem for the government [22]. Table 1 shows the Land area and population of Xi’an city in 2014.

To more effectively manage water resources, in 2003, the government of Xi’an tried to integrate water resources management [4]. Managing water resources effectively is critical to solving water shortages, and Xi’an water managers have continued to improve the management systems. Water resource management problems have been analyzed using normative analysis, resulting in development proposals and policy recommendations, and the identification of appropriate ways to manage Xi’an water resources [2,4,5,17]. For example, more attention has been paid recently to underground water conservation; a fund management system has been put into practice; and the legal system has improved. An example of such legal frameworks is *The Management Method for Water Resources in Xi’an*, *The Regulation of Groundwater in Xi’an*. Although different methods have been used, there is still not a very good effect for water resources management. Water resources are a kind of ecosystem services, and the management of water resources is to allow people to obtain better benefits. People have also been paying more attention to sustainable water use, and emphasizing ecological effects. Water resource management and the associated ecological effects are inseparable [23]. The concept of an ecological system is applied to the management of water resources, which can directly produce the results related to ecological effects. The good development of ecology will promote the management and allocation of water resources. Therefore, we apply ecosystem services to water resources management, providing a new method for decision makers to ensure the sustainable use of water resources.

### 2.2. Selecting Ecosystem Services Indicators in Different Xi’an Districts

Ecosystem service indicators for this study were determined based on the classification standard developed by MA [8]. After this determination, we evaluated ecosystem service levels in each district and county. Ecosystem services consist of four main factors, provisioning services, regulating services, cultural services, supporting services. Selecting ecosystem service indicators plays an important role in assessing ecosystem services, and should follow these principles [15]:

(1) Ecosystem services indicators should have a wide range, which means selecting as many different types of ecosystem services as possible, to increase the reliability of the results.
(2) Ecosystem services indicators should serve as a typical indicator of ecosystem services in a way that shows trends and characteristics.
(3) Each index should be relatively independent, to avoid incorrectly expressing a district’s ecosystem service due to repetitive scoring.
(4) Selected indicators should facilitate public understanding and communication with decision makers.

This paper adopted the ecosystem service framework developed by the Economics of Ecosystems and Biodiversity (TEEB); we selected our performance measures from a master list of potential ecosystem service indicators compiled from the Millennium Ecosystem Assessment [24]. These publications reflect the most recent and comprehensive assessment frameworks [25]. 

Agricultural production and water interception in ecosystem services are of the most closely relationship with people. They ensure the survival of people. Carbon sequestration and soil retention are very important ecological indicators in Arid Areas. Xi’an is a big city in Western China. With the exception of provisioning services and regulation services, cultural services is also an important measure. According to the specific situation of people’s cultural and entertaining life in Xi’an, we choose three indicators including forest park, recreational opportunity, residential properties near the river. Based on the realities in specific districts and counties, and the grounding principles above, this study used seven indicators of ecosystem services: food and fiber, freshwater, carbon sequestration, moderation of extreme events, spirituality and sense of place, recreation and physical and mental health, and aesthetic appreciation and cultural inspiration. Every index has a quantifiable indicator, which considers provisioning, regulating, and cultural service functions (Table 2). 

Given these indicators, this research mapped the actual values associated with the different indicators across all the districts and counties to more easily quantify performance. These indicators were divided into five categories (lower, low, medium, high, higher), and were marked with different colors from lowest to the highest. 

### 2.3. Data Source

The land use map was interpreted using cloud-free 30 m × 30 m resolution Landsat™ remote sensing images downloaded from the China Geospatial Data Cloud (http://www.gscloud.cn). Climatic data collected from the meteorological department included: rainfall, temperature, evaporation, and solar radiation. Digital Elevation Models with a 30 m × 30 m resolution were obtained from the National Geomatics Center of China. The soil types of Xi’an were obtained from the Soil Survey Office of Shaanxi Province. Social and economic data were obtained from the *Statistical Yearbook of Xi’an* and the *Statistical Yearbook of Shaanxi Province* [26]. Table 3 provides details about the data sources.

### 2.4. Analysis Methods

#### 2.4.1. Agricultural Production

Annual grain production in each district represents food production services; study data came from the *Statistical Yearbook of Xi’an* and the *Statistical Yearbook of Shaanxi Province* [26].

#### 2.4.2. Water Interception

The water conservation of the ecosystem is acquired through interception, evaporation, absorption and storage of precipitation, including interception from canopy, holding from litter, and water storage from soil. Vegetation storage capacity is the sum of all those three parts, also known as integrated water storage capacity method [27]. The computational formula is as follows:

(1) $$Q=A\;\times \;J\;\times \;\left(R_{o}-T_{i}/P\right)$$

In this expression, *A* is area; *J* is the average annual rainfall amount; *R_o_* is the runoff coefficient of the bare land, forest land, and/or grassland; *T_i_* is the sum of runoff from the bare land, forest land, and/or grassland in experimental months; and *P* is the total rainfall in the experimental months. According to the climate characteristics of Xi’an City, runoff mainly occurred from May to October. As such, experiments were conducted during these months. 

#### 2.4.3. Carbon Sequestration and Oxygen Release

Our paper applied the Carnegie–Ames–Stanford Approach (CASA) model proposed by Potter [28] and Field [29] to calculate net primary productivity (NPP) [30]. Of the models that estimate NPP globally, the CASA ecosystem model is one of the most widely used models. It adequately addresses NPP spatial and temporal dynamics at regional to global scales [31]. NPP estimates are determined using two variables: (1) vegetation photosynthetic active radiation (APAR); (2) actual light use efficiency (*ε*). The specific calculation of APAR is as follows:

(2) APAR=(x,t)=SOL(x,t)×FRAR(x,t)×0.5

In this expression, SOL(x,t) is the total solar radiation of pixel x in t months (Unit: MJ/m^2^/mon), FRAR(x,t) is the absorption ratio of the vegetation layer to the incident light and effective radiation. The constant 0.5 is the ratio of available solar radiation for vegetation to the total solar radiation (radiation wavelength is 0.38–0.71).

The main determinants of maximum light energy utilization in real-life situations are moisture and temperature. The function is:

(3) $$\varepsilon \left(x,t\right)=T_{\varepsilon 1}\left(x,t\right)\times T_{\varepsilon 2}\left(x,t\right)\times W_{\varepsilon }\left(x,t\right)\times \varepsilon_{\max}$$

In this expression, $T_{\varepsilon 1}\left(x,t\right)$ is the stress on light energy utilization under low temperature conditions; $T_{\varepsilon 2}\left(x,t\right)$ is the stress on light energy utilization at high temperatures; $W_{\varepsilon }\left(x,t\right)$ is the influence coefficient of water stress; and $\varepsilon_{\max}$ is maximum light energy use in an ideal state.

After calculating APAR and *ε*, we can calculate NPP. The function is:

(4) NPP(x,t)=APAR(x,t)×ε(x,t)

In this expression, x is spatial location; t is time; APAR(x,t) is APAR of pixel x in t months (unit: MJ/m^2^/mon); ε(x,t) is the energy conversion rate of pixel x in t months (unit: gC/MJ).

In ecological systems, plants undergo photosynthesis, absorbing carbon dioxide and releasing oxygen based on the following chemical formula [32]:

(5) $$CO_{2}+H_{2}O\to CH_{2}O+O_{2}$$

Using this formula allowed us to calculate that for every kilogram of dry matter fixed (representing carbon sequestration), 1.63 kg CO_2_ and 1.2 kg O_2_ were released.

#### 2.4.4. Soil Conservation 

This study uses a Universal Soil Loss Equation (USLE) to estimate soil erosion, expressed as [33,34]:

(6) A=R·K·L·S·C·P

In this expression, *A* is soil erosion; *R* is the rainfall erosion force index; *K* is the soil erosion factor; *L* is the slope length factor; *S* is the slope factor; *C* is the vegetation coverage factor; and *P* represents soil conservation measures. Soil erosion includes real soil erosion (*A_r_*) and potential soil erosion (*A_p_*). The potential soil erosion does not consider the surface coverage type and land management factors, *C* = 1, *P* = 1. With that adjustment, the soil erosion is:

(7) $$A_{p}=R\cdot K\cdot L\cdot S$$

A realistic estimate of soil erosion considers all of the above factors, as follows:

(8) $$A_{r}=R\cdot K\cdot L\cdot S\cdot C\cdot P$$

Soil conservation, *A_c_*, is expressed as follows:

(9) $$A_{c}=A_{p}-A_{r}$$

#### 2.4.5. Forest Park and Recreational Opportunity

This study also counted the number of forest park and residential recreational opportunities in each county, based on the *Xi’an Statistical Yearbook* and local field conditions [15]. People often seek entertainment through activities such as going to the gym, watching movies, and singing in Karaoke Television rooms. According to the real-world circumstances in Xi’an City, we chose the numbers of Leisure Parks, gyms, cinemas, and Karaoke Television rooms as recreational opportunities. These entertainment sources serve people of all ages. The cultural ecosystem service is an important index to measure the whole ecosystem service ability of a region. We count the number of these places is to measure the ability of cultural ecosystem service.

#### 2.4.6. Residential Properties near the River

A buffer analysis was conducted for the major rivers using the computer program ArcGIS, with a 1000 m threshold. We also counted residential coverage to assess the degree of preference for living near the river setting.

### 2.5. Questionnaire 

The study’s questionnaire assessed citizen understanding of ecosystem services, and the current level of public understanding of the different indicators. To understand service level differences across the nine districts and four counties in Xi’an City, 1020 questionnaires were distributed in all districts and counties. The numbers of surveys distributed in each area was based on population distributions. The survey was divided into two parts: an Internet questionnaire and field survey. To maximize diversity, questionnaires were distributed to respondents of different genders, ages, careers, income levels, and cultures. The questions are presented in the Supplementary Materials Text S1. The survey questionnaire originally is the Chinese version, and in order to make more people to have a better understanding of questionnaire, the sentences we used are easy to understand. In the field survey, we have made some explanations for some of the investigators to ensure the validity of the questionnaire.

Online and field surveys were conducted simultaneously. The formal survey effort was preceded by a 3-day preliminary survey, to improve the questionnaire and support index selection based on investigator feedback each suggestion was considered and the questionnaire improved. The formal survey phase was conducted by 10 people over 21 days, from 20 June to 10 July 2015. A total of 433 online surveys and 587 field surveys were completed. Field survey participation was increased by rewarding participation with a small gift, improving the validity of the survey results. Local residents familiar with the local natural and cultural environment were selected to participate in the field investigation. 

We used 50 completed questionnaires to assess perceptions among experts, using the Delphi method. This investigative method involves using experts as respondents to gather information, relying on their knowledge and experience to make judgments, evaluations and predictions about survey problems. The Delphi investigation respondents included university professors and government staff working on ecosystem services. They held deep professional knowledge, primarily carrying out research in ecosystem services. The questionnaire for experts is the same as the online and field survey. We used this input to determine each index’s weight, providing a basis for evaluating the quantitative survey. Investigator expertise was important in exploring the questionnaire indices, and helped determine index weights. This was an important step to understanding the perceived services from ecosystem of each county and district.

### 2.6. The Rank of Each District

This study used reference projects related to water resources [35,36,37], each with advantages and disadvantages. Our goal was to establish a reasonable scheme to weigh different indicators, rather than focusing on one particular area. After classifying each index, points were assigned for the different levels. Using a Likert scale method, scores for different ecosystem service functions in different districts and counties were calculated and plotted on a map.

The ranking was based on two categories of scores: scores based on the investigators’ awareness of ecosystem service functions, and scores based on real data calculations in each district. This paper reports on both categories to explore the significance of water resource management.

When calculating people’s overall preferences, this study divided questionnaire answers into five categories (lower, low, medium, high, higher), assigning scores from low to high. The scores of the overall preferences were the weighted average of all indicator values, based on Likert model [38]. Two models were used; one focused on the subjects participating in the study, and one focusing on the indexing of districts and counties. These are described as follows:

(1) Score calculation model of each subject in an index system: The score calculation model for each indicator is as follows [18]:
  (10) $$S_{i}\;=\;\sum_{p=1}^{n}Q_{ip}\times C_{ip}÷M$$

In this expression, *S_i_* is a respondent’s score for question *i*, which is associated with a certain index; *p* is the *p*th alternative answer for question *i*; *n* is the number of alternative answers for question *i*; *Q_ip_* is the number of samples choosing the *p*th alternative answer in the question *i*; *C_ip_* is the assigning point of the *p*th alternative answer for question *i*; *M* is the total number of samples.

(2) Evaluation model of each districts and counties in an index system. The evaluation model of each districts and counties is as follows [18]:
  (11) $$S_{i}=W_{1}S_{1}+W_{2}S_{2}+\ldots +W_{n}S_{n}=\sum_{j=1}^{n}W_{j}S_{j}$$

Here, *S_i_* is the score of each district and county; *m* is the number of questions; *j* is question *j*; *W_j_* is the weight of the question *j*; *S_j_* is the score of respondents for question *j*. 

## 3. Results

### 3.1. The Questionnaire of the Ecosystem Services

As mentioned above, this study used a two-part survey approach, with online and field surveys. Surveys were distributed proportionally across Xi’an’s nine districts and four counties. Respondents were all ordinary people of this region, with a willingness to participate in this survey. Respondent ages ranged from 18 to 70 years old. 

**Figure 3:**
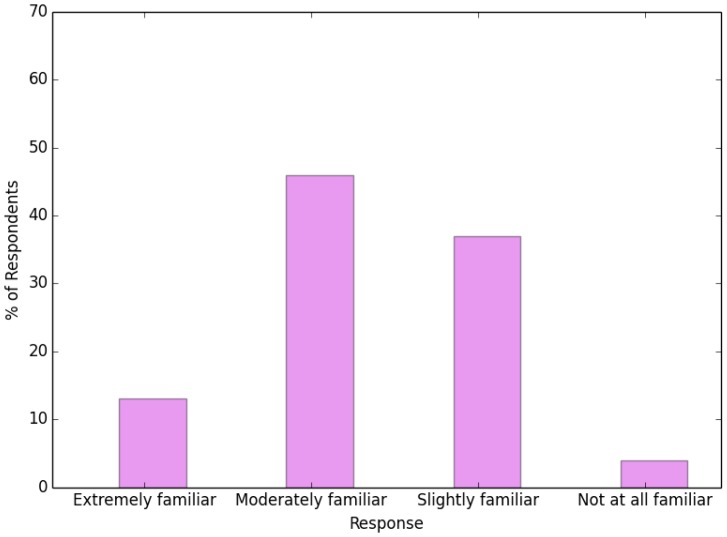
Survey results related to familiarity with ecosystem services.

This study explored respondent awareness about the level and the importance of ecosystem services, with the goal of determining each index’s weight. Figure 3 shows that the majority of respondents recognized different levels of ecosystem service functions. Only 4% of the respondents reported not really knowing any ecosystem service functions at all. On the other hand, only 13% believed that ecosystem services were extremely important. 

**Figure 4:**
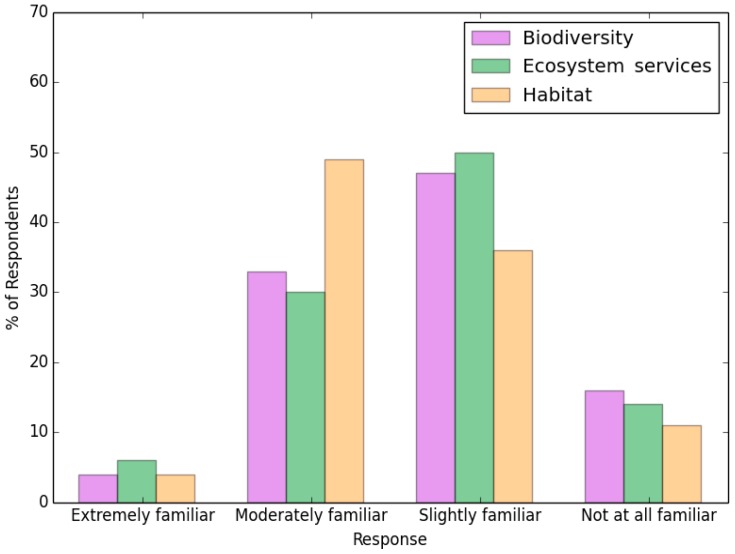
Survey results related to awareness about biodiversity, ecosystem services, and habitat.

When asked about ecosystem services, habitats, and biodiversity, there were slight differences in respondents’ understandings of these three concepts. When respondents knew more about habitats and biodiversity, they were also generally clearer about the concept of ecosystem services. Only 6% of respondents were very familiar with the concept of ecological systems, and 8% were not familiar at all (Figure 4). 

**Figure 5:**
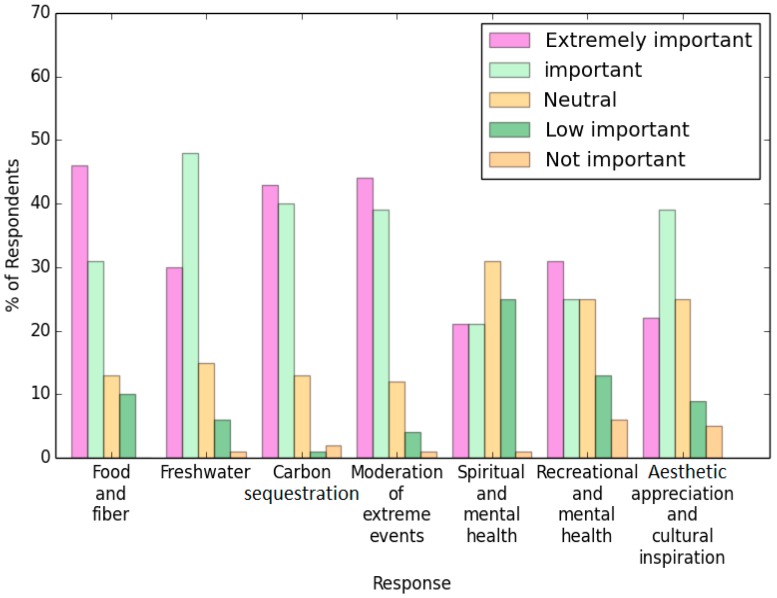
Survey results for the question: “The following are various services provided by nature in Xi’an. How important are they to YOU?”.

This study assessed seven ecosystem service indicators. When assessing people’s understanding of the importance of these indicators, the following items received the four highest weighted scores: food and fiber, freshwater, carbon sequestration, and moderation of extreme events (Figure 5).

All surveys were carried out face-to-face using the Delphi survey method; this ensured a common level of quality and effectiveness with each questionnaire and improved the credibility of each index weight. We received back 50 questionnaires, which closely aligned with our one-by-one and face-to-face interviews with experts. We established different grades for each indicator answer, scores ranged from 1 to 5. We counted the returned questionnaires, established each indicator’ score, and normalized the result. This yielded the target weights for each ecosystem service index used (Table 4). 

### 3.2. The Ability to Supply Ecosystem Services

**Figure 6:**
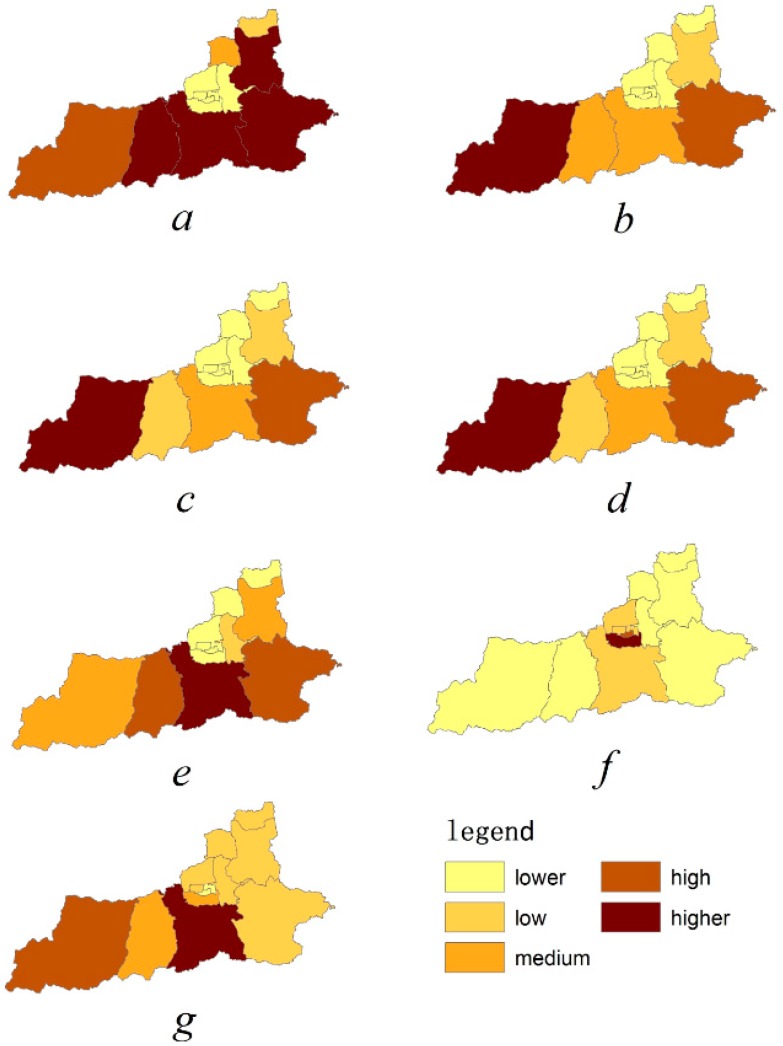
Ecosystem service supply across the 13 districts: (**a**) Food and fiber; (**b**) Freshwater; (**c**) Carbon sequestration; (**d**) Moderation of extreme events; (**e**) Spiritual and sense of place; (**f**) Recreational and mental health; (**g**) Esthetic appreciation and cultural inspiration.

Based on each index’s calculation method, we established values for each ecosystem service indicator used across regions (Supplementary Materials Table S1). Figure 6 provides the quantitative values of the ecosystem service supply capacities for Xi’an counties. We took the highest and lowest value of each index, and divided into five approximately equal intervals based on averages. The five intervals were defined as lower, low, middle, high, and higher. The following figure displays the indicators’ spatial distribution. Examining the five levels for each indicator showed that indicators and supply capacities clearly varied across counties. Food and fiber were at a higher level in four regions; the other six indicators were at a higher level only in one region. Recreational and mental health was at a lower and low level in most regions. This indicated that the supply capacity of ecological system services on this aspect was poor overall. There were no areas where ecosystem services for all seven indicators were classified at a higher level. There were also no areas where services were all classified at a minimum level; levels varied from region to region. Thus, it is essential to calculate the ecosystem services of all seven indexes across areas. 

### 3.3. Ranking of Each Area

This study ranked districts and counties based on each county’s ecosystem supply capacity (Supplementary Materials Table S1). The ranking clearly quantified different indicators in these counties; each index’s weight was achieved using questionnaire results. Each county was given a score based on the study’s indicator weightings. Points were assigned to different levels within each index: 1 point for a lower level, 2 points for low, 3 points for the middle level, 4 points for high, and 5 points for higher. We calculated the ecosystem service function scores across regions; weights were adopted based on statistical results from a Delphi survey method. Calculations were done using Likert statistical methods. 

Using Xincheng district for example, the indicator score for food and fiber was 1; freshwater was 1; carbon sequestration was 1; moderation of extreme events was 1; spiritual and sense of place was 1; recreation and physical and mental health was 2; and aesthetic appreciation and cultural inspiration was 1. Each score was multiplied by its corresponding weight, leading to a gross score for ecosystem service supply capacity. The result for that example was 1.12, based on Likert statistical methods. In this example, the contrast is not as obvious as for other cases, because of the small amounts that are enlarged 100 times, yielding and get 112 points. 

**Figure 7:**
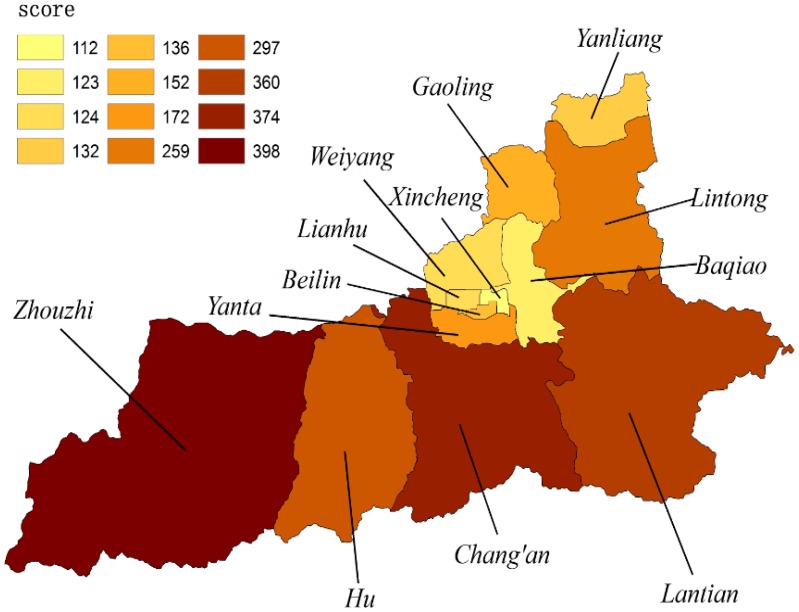
The score after being weighted for indicators.

We calculated the scores of ecosystem services supply capacity across all the areas using the same method (Figure 7). The highest score was for Zhouzhi county, at 398. This was followed by Chang’an district and Lantian county, with scores of 374 and 360, respectively. Scores decreased in sequence for Yanta district, Gaoling district, Beilin district, Yanliang district, Lianhu area, Weiyang area, and Baqiao district. Xincheng district had the lowest score of 112.

## 4. Discussion

### 4.1. Correlation between “Amount of Water Resources” and “Supply of Ecosystem Services”

**Figure 8:**
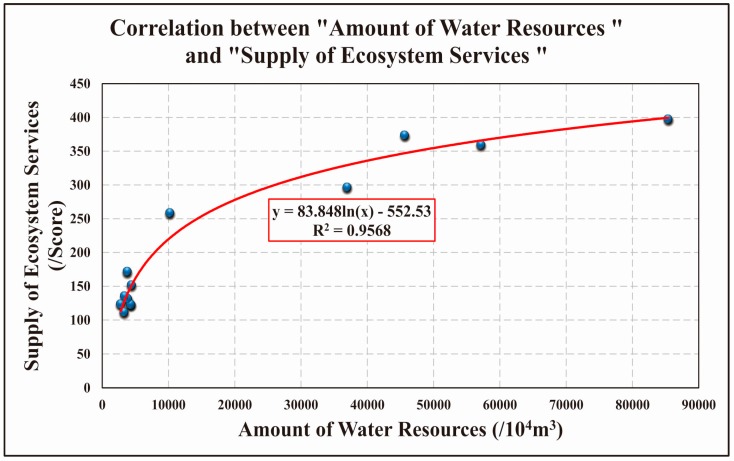
Correlation between “Amount of Water Resources” and “Supply of Ecosystem Services”.

Based on the supply capacity of ecosystem services across regions, we determined whether there was a correlation between total water resources and the supply capacity (Figure 8). There was a clear relationship between these two variables across high ecosystem services regions. The water-deficient area had a weaker supply capacity of ecosystem services; the area with more total water resources had a higher supply capacity. For example, the following regions have fewer water resources, and their supply capacity of ecosystem services are mostly under 200 points: Xincheng district, Baqiao district, Weiyang district, Lianhu district, Yanliang district, Beilin district, Yanta district, Gaolin district. Regions with more water resources have a higher supply capacity of ecosystem services. These include: Lintong district, Lantian county, Chang’an district and Zhouzhi county. There are a few of the points that are gathered together in Figure 8, representing the supply of ecosystem services and the amount of water resources are both low. These points are located in the urban area of Xi’an City, and the area is small. Therefore the supply of ecosystem services and the amount water resources are relatively small. Although this cannot be a good relationship between the supply of ecosystem services and the amount of water resources, it does not violate the cooperative relationship between them. At the high ecosystem services regions such as Lantian county, Zhouzhi county, and Chang’an district, the relationship between supply of ecosystem services and the amount of water resources is more obvious. Above all, it’s feasible to take the supply of ecosystem services into consideration when conducting the management of water resources. 

This article shows contrasting correlations between the amount of water resources and supply of ecosystem service in the 13 regions. The results show that these two dimensions are significantly correlated, with a correlation coefficient of R^2^ = 0.9568. Figure 8 shows a significant synergistic relationship between the total water resources and supply capacity of ecosystem services. This also provided an important evidence that using ecosystem service capacities can guide the design of water management policy, leading to more scientifically based decisions in special districts.

### 4.2. Combining Water Resource Management with Ecosystem Services

Water management has received significant attention in regions of China, as people have tried to combine water management with energy management [39], economy, climate [40], water resource quantities, and regional environmental management. This study combined a consideration of water management with ecosystem services. Using the case study of Xi’an, we related ecosystem service levels with water resources and management. Ecosystem services include economic, climatic, environmental, spiritual, and other factors. As such, it differs from the practice of considering only a single factor. 

Other researchers have tried to study ecosystem service levels of different catchments, by differentiating catchment areas to provide reference points for water resource management. In contrast, this study used administrative divisions to provide more convenient reference points. Government water management policies are very important for water resources management, and require balancing human needs for water resources and harmonious relationships between humans and nature [41]. This study built a framework to introduce the concept of ecosystem services into water resource management, providing a new reference for government decision-making. 

**Figure 9:**
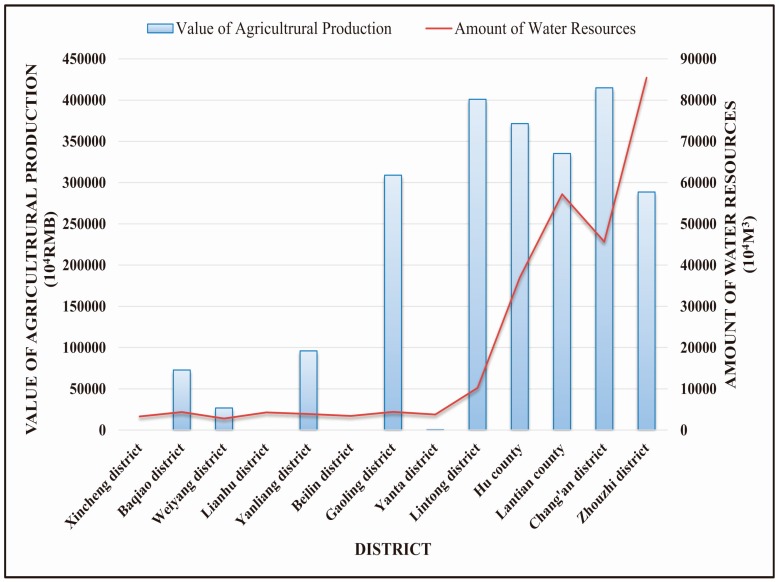
Correlation between “Amount of Water Resources” and “the Value of Agricultural Production”.

This study framework has a number of advantages. For example, it investigated stakeholder preferences, allowing for reductions in the contradictions that emerge in developing water resources management policy. For example, traditional water resources management considers the problem of needing sufficient water for agricultural irrigation, highlighting grain production as an important factor. This paper contrasted the value of agricultural production with peoples’ overall preferences (Figure 9). Based on this collaborative relationship, we found that ecosystem service values provide more extensive reference points for water resources management, and offer possible directions for reform. This study highlights that a single index offers only a partial view. For example, in a Xincheng district, the Beilin district, and the Lianhu district, there is no expressed value from agricultural production. However, these three locations cannot be ignored when formulating water resources management policies. These three places are located in Xi’an’s downtown area, where there is higher domestic water demand and a synergistic relationship with people’s overall preferences.

A water resource management approach that is based solely on agricultural needs cannot adapt to all the needs of Xi’an faces with respect to water resources management development. In recent years, China has regulated water resource management allocations primarily according to water resources in each region. So far, there have been no areas that can combine the supply capacity of ecosystem services with water resources management. In our study, we find there is strong credibility in applying this approach. 

### 4.3. The Advantages and Disadvantages of the Questionnaire Survey

Survey validity must be considered when investigating the importance of ecosystem service indicators and determining indicator weights. Questionnaire surveys are a more direct and effective method. Based on certain proportions in responses, we can assess common levels of understanding about the ecosystem. Moreover, based on this shared understanding, we can determine the weight of each ecosystem service index. This is a common, accepted, and mature research method: researchers determine an index weight using a survey, and obtain target scores. 

When choosing respondents, it is hard to distinguish specific factors that may influence survey results, such as age, geography, education level, and gender. In this study, for example, we found that people with more education are more familiar with ecosystem services. Different respondents from different areas and different careers, perceive the index differently. For example, farmers report that irrigation and grain are more important than other indicators. People who work in the fields of ecology and environment report paying attention to all indicators. People living in the city report that spiritual experiences are more important, and report that entertainment and leisure are also important. Interviewee differences do not allow for exact statistics, and there is no mode to consider. As such, while survey methods are useful tools, their fuzziness cannot be ignored, and require that we consider ways to further increase accuracy. 

In addition, this paper has noted questionnaire inaccuracies. The questionnaire content only assessed items with a close relationship with human life, such as food, water, and moderation of natural disasters. Questionnaire design should also include four aspects of ecosystem services, with as much richness as possible. People’s understanding is not the same for different problems, so the uncertainty of the problem will directly affect the results of the questionnaire.

Because the survey time was only 21 days, we distributed only 1020 valid questionnaires. To increase survey result accuracy, the number of questionnaires would need to be expanded, and combined with the population, economy, distribution and other factors in each area. In our survey, we only considered demographic factors, based on the population in each county to proportionally distribute questionnaires. In future studies, we should consider important factors, such as age and educational level, in distributing the questionnaires. For example, age could be divided into five stages, each with a target sample size of 300 people, with a target level of educational diversity. Adding a variety of factors to the questionnaire may also yield more accurate results. 

### 4.4. Study Limitations

Integrating ecosystem services with water resources management is an innovative approach in China, but disadvantages remain. This paper has noted possible inaccuracies with respect to the questionnaire; more factors may improve accuracy. Further, while there are many ecosystem service indicators, this paper selected only the seven most common ones. While the seven indicators do reflect different dimensions, some indicators fail to accurately represent county-level ecosystem services. Indicator selection directly affects ecosystem service levels; as such, more indicators should be considered to improve accuracy. 

## 5. Conclusions

The changing world climate and environmental changes highlight problems and needs associated with water resources management, including reductions in fresh water resources and the growing demand for water. Reasonable water resource management is an effective way to solve water resource shortages. In this paper, the ecosystem services are used in water resources management, through a series of methods and models, include questionnaires, indicators, correlation analysis and so on, to explore the relationship between ecosystem services and water resources. Traditional water resources management considers the problem of needing sufficient water for agricultural irrigation, highlighting grain production as an important factor. It is found that the area with high grain yield is not necessarily the area with abundant water resources. Simply considering the water resources management of grain production cannot meet the needs of the development of the city. Based on the supply capacity of ecosystem services across regions, we determined whether there was a correlation between total water resources and the supply capacity and found that there was a clear relationship between these two variables across regions. The water-deficient area had a weaker supply capacity of ecosystem services; the area with more total water resources had a higher supply capacity. The amount of water resources can be allocated according to the level of ecosystem services in the area. In areas with low ecosystem service capacity, we can consider more water resources to ensure the development of the area. This study provides a framework for combining the concept of ecosystem services and water resource management, providing government policy specialists new water management methods. 

The framework developed in this study advances progress in water resources management approaches. It can be used to consider a single factor solution within a more comprehensive multi-factor evaluation. Differences in ecosystem service functions directly reflect differences between environments in different areas. If water resources management is simultaneously managed with ecosystem services, regional environmental and ecological problems can also be considered. By selecting ecosystem service indicators, and investigating these indicators through a questionnaire, we obtained weighted information and assess stakeholder preferences, directly supporting water resources management. Relationships among different indicators were easy to identify, based on the quantitative weighing of the indexes and data from across the various regions. This framework provides a promising new direction for water resources management. 

## Figures and Tables

**Table 1:** Land area and population of Xi’an city in 2014.

District and County	Total Population (Million)	Land Area (km^2^)
Xi’an city	815.29	10,097
Xincheng district	50.80	30
Beilin district	71.02	23
Lianhu district	65.75	38
Baqiao district	53.60	325
Weiyang district	58.70	264
Yanta district	83.25	151
Yanliang district	26.26	245
Lintong district	71.85	916
Chang’an district	105.93	1589
Lantian county	65.45	2006
Zhouzhi county	68.79	2945
Hu county	60.98	1279
Gaoling county	32.91	285

**Table 2:** Ecosystem service suppliers.

Categories	Indicators	Performance Measure
Provisioning Services	Food and fiber	Agricultural production (tons/year)
Provisioning Services	Freshwater	Water interception (tons/year)
Regulating Services	Carbon sequestration	Carbon sequestration and oxygen release (tons of carbon/year)
Regulating Services	Moderation of extreme events	Soil retention (tons/year)
Cultural Services	Spiritual and sense of place	Forest Park (counts)
Cultural Services	Recreational and mental health	Recreational opportunity (counts)
Cultural Services	Aesthetic appreciation and cultural inspiration	Residential properties near the river (ha)

**Table 3:** Data and data resources.

Required Data	Agricultural Production	Water Interception	Carbon Sequestration and Oxygen Release	Soil Retention	Forest Park and Recreational Opportunity	Residential Properties near the River	Total Amount of Water Resources	Data Source
Land-Use Map		▲		▲		▲		Landsat (TM)
DEM				▲				Topographic Map of 1:50,000
Soil Type				▲				Agricultural Sector
Rainfall				▲				Meteorological Department
Temperature			▲					Meteorological Department
Evaporation			▲					Meteorological Department
Solar Radiation			▲					Meteorological Department
Administrative Map		▲	▲			▲		Civil Affairs
Grain Production	▲							Statistical Yearbook
Fiber Production	▲							Statistical Yearbook
Places of Entertainment					▲			Statistical Yearbook
Population						▲		Statistical Yearbook
Water Resources							▲	Statistical Yearbook

▲ represents the sources of data for different indicators and water resources.

**Table 4:** The weight for each ecosystem services derived from the survey data.

Ecosystem Service	Performance Measure	Weight (%)
Provisioning Services		
Food and fiber	Agricultural production (tons/year)	20
Freshwater	Water interception (tonnes/year)	13
Regulating Services		
Carbon sequestration	Carbon sequestration and oxygen release (tonnes of carbon/year)	17
Moderation of extreme events	Soil retention (tons/year)	15
Cultural Services		
Spiritual and sense of place	Forest Park (counts)	11
Recreational and mental health	Recreational opportunity (counts)	12
Aesthetic appreciation and cultural inspiration	Residential properties near the river (ha)	12

## Supplementary Materials

The following are available online at www.mdpi.com/1660-4601/13/11/1169/s1. Text S1: Survey questions regarding respondents’ awareness of the benefits provided by nature, familiarity with the concept of ecosystem services, and the importance of the ecosystem services to the respondents, Table S1: The consequence table used for the Xi’an.
