# Supplementary material for: Ecosystem Services Insights into Water Resources Management in China: A Case of Xi’an City
Source: Int J Environ Res Public Health. 2016 Nov 24;13(12):1169. doi: 10.3390/ijerph13121169 (PMC5193282; doi:10.3390/ijerph13121169)
Supplement: Supplementary file 1 [file ijerph-13-01169-s001.pdf]

Supplementary Materials: Ecosystem Services Insights into Water Resources Management in China: A Case of Xi’an City

Jingya Liu, Jing Li, Ziyi Gao, Min Yang, Keyu Qin and Xiaonan Yang

Supplmentary Text S1. Survey questions regarding respondents’ awareness of the benefits provided by nature, familiarity with the concept of ecosystem services, and the importance of the ecosystem services to the respondents.

Questions for Figure 2. Have you ever thought of nature in regards to the benefits and services outlined in previous question? Please state your level of awareness prior the survey.

Extremely aware ☐ Moderately aware ☐ Slightly aware ☐ Not at all aware ☐

Questions for Figure 3. How familiar are you with the following terms?

(a) Biodiversity

Extremely familiar ☐ Moderately familiar ☐ Slightly familiar ☐ Not at all familiar ☐

(b) Ecosystem services

Extremely familiar ☐ Moderately familiar ☐ Slightly familiar ☐ Not at all familiar ☐

(c) Habitat

Extremely familiar ☐ Moderately familiar ☐ Slightly familiar ☐ Not at all familiar ☐

Questions for Figure 4. The following are various services provided by nature in Xi’an. How important are they to YOU?

(a) Water for irrigation

Extremely important ☐ Moderately important ☐ Neutral ☐ Low importance ☐ Not important ☐

(b) Food: Fruit, grain, vegetable, etc.

Extremely important ☐ Moderately important ☐ Neutral ☐ Low importance ☐ Not important ☐

(c) Trees store carbon: healthy trees trap carbon from the atmosphere and help reduce air pollution

Extremely important □ Moderately important □ Neutral □ Low importance □ Not important □

(d) Moderation of natural disasters: natural vegetation can reduce the risk of extreme flooding, etc.

Extremely important □ Moderately important □ Neutral □ Low importance □ Not important □

(e) Spiritual experience and sense of place: be at one with nature and sense of belongingness

Extremely important □ Moderately important □ Neutral □ Low importance □ Not important □

(f) Recreation, mental and physical health

Extremely important □ Moderately important □ Neutral □ Low importance □ Not important □

(g) Aesthetic appreciation and inspiration for culture, art and design

Extremely important □ Moderately important □ Neutral □ Low importance □ Not important □

(h) Maintenance of biodiversity: maintaining the richness and variety of nature

Extremely important □ Moderately important □ Neutral □ Low importance □ Not important □

Table S1. The consequence table used for the Xi'an.

Objective	Food and Fibre	Freshwater	Carbon Sequestration	Moderation of Extreme Events	Spiritual and Sense of Place	Recreational and Mental Health	Aesthetic Appreciation and Cultural Inspiration	Water Resources
**Performance Measure**	**Agricultural Production (Tons/Year)**	**Water Interception (Tonnes/Year)**	**Carbon Sequestration and Oxygen Release (Tonnes of Carbon/Year)**	**Soil Retention (Tons/Year)**	**Forest Park (Counts)**	**Recreational Opportunity (Counts)**	**Residential Properties near the River (ha)**	**Total Amount of Water Resources (10^4 m^3)**
Xincheng district	0	11,239,500	2,298,240	100	0	10	2360	3321
Beilin district	0	4,353,680	876,819	31	0	1	192	3417
Lianhu district	0	186,370	42,371.4	14	0	18	10,141	4271
Baqiao district	72,800	1686.6	2998.57	70	0	85	29,077	4385
Weiyang district	26,800	2741.82	5584.37	58	224	62	17,594	2798
Yanta district	600	5,072,330	1,038,150	9	1572	11	3031	3782
Yanliang district	96,000	799.8	4789.55	46	13,074	120	47,052	3891
Lintong district	401,000	7,154,690	1,462,140	19	2432	40	23,862	10,243
Chang'an district	414,900	449,551	77,975.8	43	115	15	6636	45,663
Lantian county	335,300	745,414	134,999	37	0	20	12,668	57,177
Zhouzhi county	288,600	799,947	167,138	48	0	34	22,838	85,431
Hu county	371,500	2,803,800	568,296	51	22,855	56	24,691	36,983
Gaoling county	209,000	730,521	142,528	98	23,607	8	173	4430

© 2016 by the authors. Submitted for possible open access publication under the terms and conditions of the Creative Commons Attribution (CC-BY) license (http://creativecommons.org/licenses/by/4.0/).
